# Atrial fibrillation gut syndrome: severe gastroparesis with pyloric spasm following radiofrequency catheter ablation of drug resistant symptomatic atrial fibrillation: a case report

**DOI:** 10.1186/s13256-023-03829-w

**Published:** 2023-04-18

**Authors:** Lakshmi Kannan, Anfal Fahim

**Affiliations:** 1 Division of Nephrology, Department of Internal Medicine, Pikeville Medical Center, 911 Bypass Rd, Pikeville, KY 41501 USA; 2 Department of Internal Medicine, Pikeville Medical Center, Pikeville, KY 41501 USA

**Keywords:** Atrial fibrillation, Gastroparesis, Radiofrequency catheter ablation, Botulinum toxin

## Abstract

**Background:**

Atrial fibrillation is the most common cardiac arrhythmia, and cardiac ablation is one of the treatment modalities for persistent symptomatic atrial fibrillation. Gastroparesis is a rare complication of radiofrequency catheter ablation for atrial fibrillation, which may be associated with high morbidity.

**Case presentation:**

We present a 44-year-old Caucasian male with persistent atrial fibrillation who presented with nausea, vomiting, bloating, and constipation after radiofrequency catheter ablation. He was found to have gastroparesis due to pyloric spasm that was treated with botulinum toxin injection.

**Conclusion:**

This case signifies the importance of identifying gastric complications after radiofrequency catheter ablation atrial fibrillation, and the need for prompt diagnosis and treatment of gastroparesis with botulinum toxin injection.

## Introduction

Gastroparesis, defined as delayed gastric emptying without mechanical obstruction of the stomach, is a rarely reported complication of catheter ablation for atrial fibrillation (AF) [1]. There are no diagnostic criteria, hence imaging studies such as gastrointestinal series and endoscopy are required. A majority of the cases of gastroparesis are caused by diabetes, drugs such as anticholinergics, opioids, tricyclic antidepressants, and phenothiazines, and postsurgical complications. A prospective observational study, the Atrial Fibrillation Gut Study by Lakkireddy *et al.* in 2015 [2] showed that new-onset esophageal dysmotility and delayed gastric emptying time were observed in 48% of patients.

Here, we present a rare case of a patient with gastroparesis with pyloric spasm after radiofrequency catheter ablation (RFCA), who recovered with botulinum toxin injection.

## Case presentation

A 44-year-old Caucasian male with history of idiopathic cardiomyopathy, hypertension, atrial fibrillation, and depression presented to the hospital with nausea, vomiting, and decreased appetite for 2 weeks. Since discharge after undergoing radiofrequency catheter ablation for atrial fibrillation 2 weeks ago, he has been having nausea, multiple episodes of vomiting, bloating, and constipation. The patient denied any fever, cough, shortness of breath, abdominal pain, or recent use of antibiotics or nonsteroidal anti-inflammatory drugs (NSAIDs). His only medications at the time of admission were omeprazole, amiodarone, ramipril, and Eliquis. Notably, the patient had undergone direct current cardioversion (DCCV) of atrial fibrillation 8 months previously and cardiac ablation 5 months previously due to persistent symptoms.

On the day of admission, physical examination revealed soft, distended, and nontender abdomen. The patient was afebrile with a blood pressure of 112/81 mm Hg, pulse of 74 beats per minute (regular), and respiratory rate of 20 breaths per minute. Laboratory work is shown in Table 1.

**Table 1:** Laboratory work-up at the time of admission

Laboratory test	Result	Reference range
Hemoglobin	12.2 g/dL	11.6–15 g/dL
Hematocrit	38%	35.5–44.9%
White blood cells	4700/mL	4500–11,000/mL
Platelets	206,000/mm^3^	150,000–450,000/mm^3^
Sodium	138 mEq/L	135–145 mEq/L
Potassium	4.0 mmol/L	3.6–5.2 mmol/L
Calcium	9.2 mg/dL	8.5–10.2 mg/dL
Magnesium	1.8 mg/dL	1.7–2.2 mg/dL
Phosphorus	3.8 mg/dL	2.8–4.5 mg/dL
CO_2_	24 mEq/L	22–29 mEq/L
Blood urea nitrogen	21 mg/dL	6.0–23.0 mg/dL
Creatinine	1.10 mg/dL	0.5–1.4 mg/dL
International normalized ratio (INR)	0.8	0.84–1.21
Aspartate aminotransferase (AST)	20 IU/L	5–40 IU/L
Alanine aminotransferase (ALT)	18 IU/L	5–40 IU/L
Amylase, lipase	Negative	40–140 U/L

**Fig. 1:**
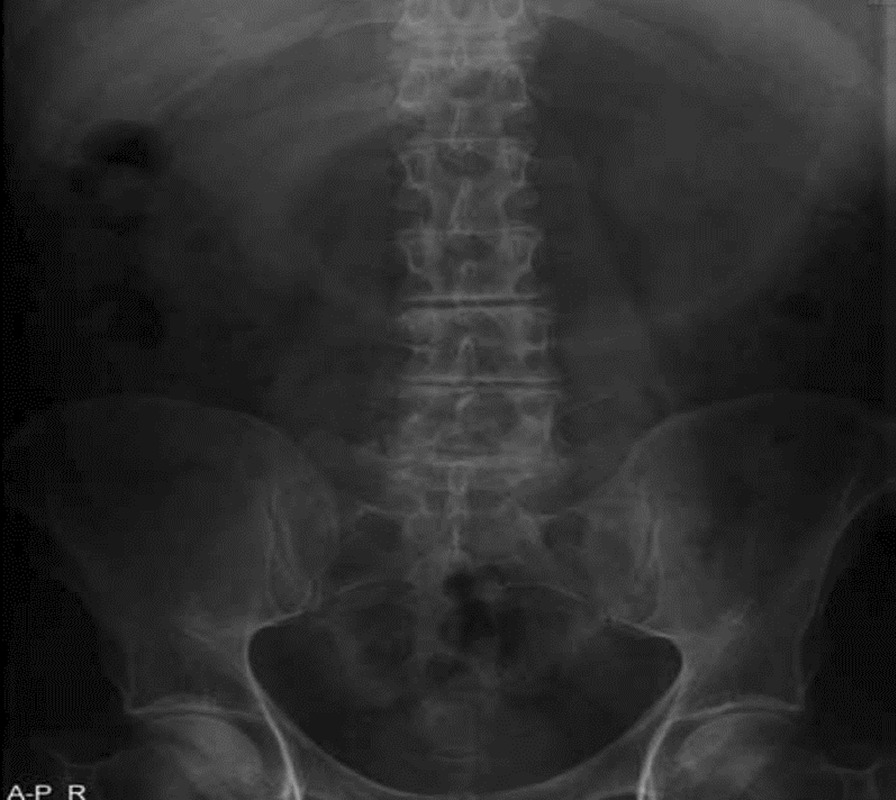
Abdominal X-ray showing distended stomach

He had an abdominal X-ray (AXR) that showed a distended stomach as shown in Fig. 1.

**Fig. 2:**
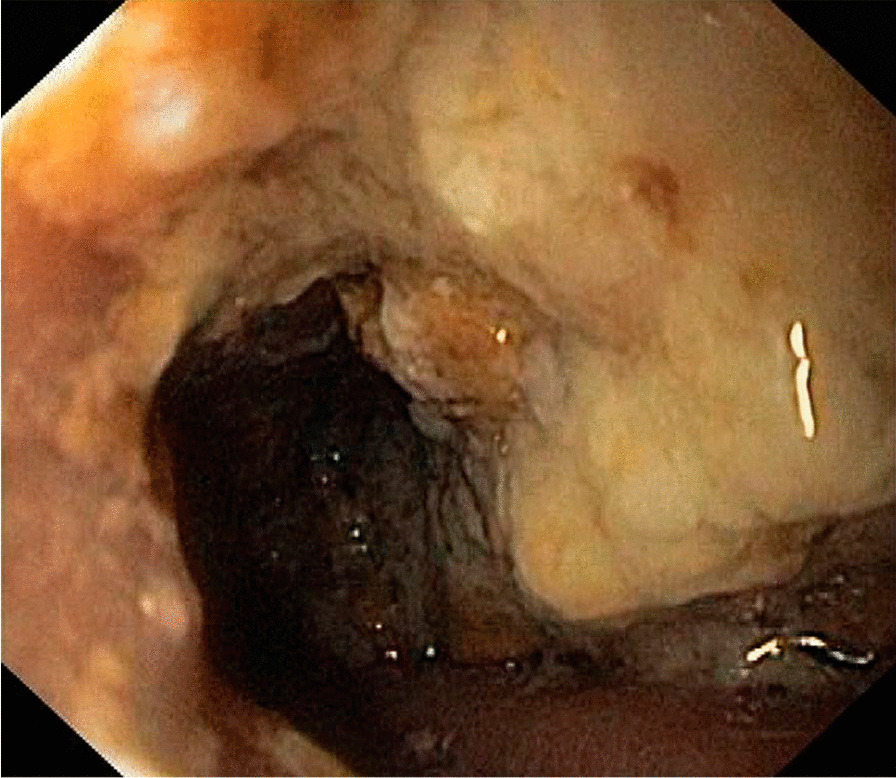
Esophagogastroduodenoscopy for botulinum toxin injection in the four quadrants as shown

The patient was admitted and he underwent an esophagogastroduodenoscopy (EGD), which showed a moderate amount of food residue in the gastric body and fundus with a hypertensive pylorus, but the scope was able to traverse it (Fig. 2). He was discharged home on a low-residue diet, metoclopramide, and pantoprazole.

**Fig. 3:**
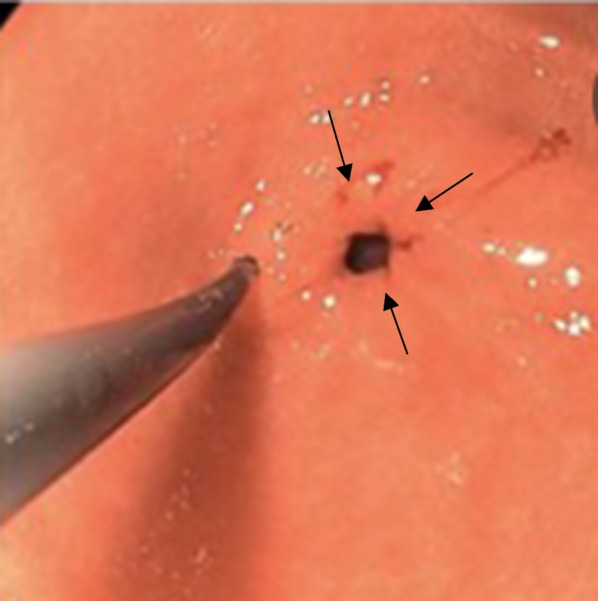
Endoscopy with botulinum toxin injection in the four quadrants of the pylorus. Arrow points to the four quadrants where botulinum toxin was injected

A repeat EGD was done a week later as symptoms were not relieved, which again showed a large amount of solid food in the stomach and the antrum could only be partially evaluated. The procedure was aborted due to the high risk of aspiration. EGD was repeated the next day under general anesthesia with endotracheal intubation. It showed spastic pyloric channel, and 10 units botulinum toxin was injected into the four quadrants of the pylorus as shown in Fig. 3. The pylorus was observed for 5 minutes for response, and eventually the scope was able to traverse the pyloric channel into the duodenal bulb.

## Discussion

Vagus nerve injury is one of the least known complications of radiofrequency catheter ablation for atrial fibrillation. A retrospective study conducted by Knopp *et al.* in 2014 [3] recruited 425 patients with symptomatic AF who underwent left atrial RFCA and subsequent EGD 1–3 days after the procedure, and who showed pathological gastrointestinal findings that included gastric erosion (22%), esophageal erythema (21%), gastroparesis (17%), thermal esophageal lesions, and reflux esophagitis.

The largest study to date by Park *et al.* involving 5380 patients undergoing AF ablation reported a 0.2% incidence of gastroparesis [4].

Gastroparesis presenting as retention of food residue in the stomach is caused by damage to the vagal nerve fibers surrounding the distal esophagus, and because of its close proximity to the posterior wall of the left atrium and left pulmonic veins [5]. The right and left vagus nerves form an anterior and posterior plexus called the periesophageal plexi that supply the stomach and the pyloric sphincter. The mechanism of injury in atrial fibrillation gut syndrome is multifactorial but could be from direct thermal injury, regional nervous system injury, or microvascular complications such as edema and microhematomas [6].

The vagus nerve injury is usually transient and symptoms include abdominal pain, progressive bloating, and early satiety [7]. Treatment includes initially ruling out mechanical causes such as gastric outlet obstruction, then using prokinetics, low residue diet, botulinum toxin injection, and in severe cases, gastric bypass surgery or total gastrectomy.

There is no clear consensus on the prevention strategies, but studies have reported using less thermal energy when ablating the posterior wall of the left atrium, monitoring of luminal esophageal temperature, using intraesophageal balloon cooling measures, and mechanical displacement of the esophagus during ablation [1, 8, 9].

In our patient, despite the initial conservative management with a low residue diet, pantoprazole, and metoclopramide, the symptoms persisted leading to weight loss and malnutrition, so botulinum toxin was used. Botulinum toxin is a bacterial neurotoxin that, at low doses, inhibits calcium-dependent release of acetylcholine from cholinergic nerve terminals and, at higher doses, inhibits smooth muscle contraction [10].

Gastroparesis after RFCA is a rare complication that should be suspected when patients complain of nausea, constipation, and abdominal bloating. A trial of prokinetics and proton pump inhibitors with a low-residue diet can be given initially. If there is no improvement in symptoms, one may consider esophagogastroduodenoscopy.

## Conclusion

This case highlights that gastrointestinal complications should be anticipated after RFCA, which can be conservatively managed in some cases, while in others it may be more challenging and require esophagogastroduodenoscopy. Botulinum toxin injection can be used when pyloric spasm is found.

## Data Availability

All data generated or analyzed during this study are included in this article.
